# Outreach Syphilis Testing Services by Different Health Providers to Female Sex Workers in Southern China

**DOI:** 10.1371/journal.pone.0060626

**Published:** 2013-04-23

**Authors:** Xiang-Sheng Chen, Yue-Ping Yin, Guo-Gu Liu, Wan-Hui Wei, Hong-Chun Wang, Yuan-Lin Yu, David C. Mabey, Rosanna W. Peeling

**Affiliations:** 1 National Center for STD Control, China Center for Disease Control, Nanjing, China; 2 Institute of Dermatology, Chinese Academy of Medical Sciences, Nanjing, China; 3 Health Bureau of Liuzhou City, Liuzhou, China; 4 London School of Hygiene and Tropical Medicine, London, United Kingdom; University of Illinois at Chicago, United States of America

## Abstract

Health providers have played important roles on delivering prevention and care services to control syphilis in China. The current study was aimed to evaluate the performance of different health providers in providing outreach syphilis testing services to female sex workers (FSWs). The current study carried out during April to August 2009 in Liuzhou was aimed to investigate the services delivered by two different types of clinics in China. A total of 1,808 FSWs recruited from sex work venues were included in the study. Prevalence of positive syphilis test (6.4%) among FSWs accessed by the local center for disease control outreach teams (CDC teams) was significantly lower than that (9.3%) among FSWs accessed by the local reproductive health hospital outreach teams (RHH teams). As compared with CDC teams, RHH teams had more FSWs to be successfully referred to the designated STD clinics for further syphilis confirmation and intervention (85.7% vs. 26.7%, P<0.001). These findings indicate that RHH teams may be more efficient than CDC teams to provide outreach-based services to FSWs. Participation of the reproductive health providers or other medical facilities in outreach services to FSWs should be considered in developing intervention programs in China.

## Introduction

Syphilis, a chronic infectious disease caused by the spirochete *Treponema pallidum* and usually transmitted through sexual contact or from mother to baby, has made a strong comeback in the form of a syphilis epidemic in China at a rate faster than any other country since 1980s [1]. Female sex workers (FSWs) have been considered as key populations which facilitate increase of the epidemic and likely to determine how fast the epidemic will spread from high risk groups to the general population. Behavioral interventions focused on health education and condom promotion have been introduced nationwide as the national public health strategies in combination with HIV control program to curb the syphilis epidemic in China [2]. However, these traditional approaches of focusing on behavioral interventions is far from enough for responding the current challenges facing China as the syphilis epidemic evolves. New syphilis epidemic needs to be met by novel and responsive public health approaches to marry behavioral and biomedical interventions. Introducing free syphilis screening into STD clinic care, outreach services and HIV testing practice, and providing free or low-cost treatment and intervention services to patients infected with syphilis at clinics have been recommended as core components in syphilis control program in China [3]. In this paper, the present study was aimed to evaluate the efficiency of syphilis screening and referral services delivered by two different types of clinics in a city in Southern China.

## Methods

The study was conducted during April to August 2009 in a middle-size city (Liuzhou) in Guangxi Autonomous Region. Two health facilities, i.e., the Liuzhou Center for Disease Control (CDC) and the Liuzhou Erkong Hospital (a reproductive health hospital, RHH) were assigned by the local health authority to provide on-site interventions at sex work venues in the specific geographic areas in the city through their outreach teams (CDC teams and RHH teams). Erkong Hospital set up the outreach teams consisting of the doctors who had clinical background and were able to provide reproductive health counseling in addition to public health interventions. FSW outreach programs included regular visits to FSW settings to conduct condom promotion, sexual health education, and risk reduction counseling developed according to the national guidelines. As part of this study, free rapid syphilis testing (RST) was offered to all FSWs at sex work venues visited by the outreach teams. Eligibility of FSWs to participate in the study included age>16 years; ability to give an informed consent; and having provided commercial sex for money or goods within the previous three months. Following ethical review by the Chinese Academy of Medical Sciences Institute of Dermatology in Nanjing, all eligible patients who agreed to participate in the study were interviewed with a brief questionnaire and invited to take an on-site RST. Those who were willing to be tested had whole blood or finger prick blood collected for RST. All blood specimens were tested in outreach settings with the commercially available RST kits (Wantai anti-TP Antibody Rapid Test, Wantai Biological Pharmaceutical Co., Ltd, Beijing, China). This RST tests have shown a good performance in sensitivity and specificity of 95.1% and 95.8%, respectively, as compared with *Treponema pallidum* particle agglutination (TPPA) (Yin YP, unpublished data). The outreach team members were responsible for conducting tests, interpreting the test results, and informing the test results. Confidentiality of testing results was ensured when the FSWs were informed of their results. All FSWs with positive RST were referred by an outreach team member to the designated STD clinics for further diagnosis and treatment.

Proportion, with 95% confidence intervals (CIs), was measured. We used chi-squared (χ^2^) test to compare the proportions and Student's t test to compare the means between data from the two kinds of outreach teams. Factors with significance level of p<0.10 in univariate analysis were included in multivariate logistic regression model to explore the association of indicators with health provider. Adjusted odds ratio (AOR) and its 95% CI were estimated. Values of p<0.05 were considered statistically significant. Statistical analysis was performed using SPSS (version 18.0 for Windows; SPSS Inc., Chicago, IL) and MedCalc for Windows (version 11.1, Mariakerke, Belgium).

## Results

Out of 1,808 FSWs recruited from different types of sex work venues, 781 (43.2%) came from entertainment venues (karaoke bars, or hotels) and 1,027 (56.8%) from other venues (hair salons or barber shops, massage parlors, foot bathing shops, roadside shops, and public outdoor places). The differences between FSWs accessed by CDC and RHH teams were not statistically significant in terms of the average age (25.1±7.3 *vs.* 25.4±6.6 years, p = 0.43), the proportion from the entertainment venues (42.5% *vs.* 44.6%, p = 0.38), or the history of syphilis infection (2.8% *vs.* 2.0%, p = 0.41). The prevalence of RST positivity (5.8%) among the FSWs accessed by CDC teams was significantly lower than that (8.5%) by RHH teams (p = 0.03), Table 1.

**Table 1:** Background information of FSWs and outcomes of RST-based services by health provider.

Variable	Health provider		P value
	CDC teams	RHH teams	
Number of FSWs accessed	1200	605	
Background characteristics			–
Age in years, mean±SD	25.1±7.3	25.4±6.6	0.43
Entertainment-based FSWs, % (95% CI)	42.5 (39.7–45.3)	44.6 (40.7–48.6)	0.38
History of syphilis infection, % (95% CI)	2.8 (2.0–3.9)	2.0 (1.1–3.5)	0.41
Prevalence of RST positivity, % (95% CI)	5.8 (4.5–7.3)	8.5 (6.5–11.0)	0.03
Outcomes of RST-related services			
Willingness to get RST, % (95% CI)	92.8 (91.2–94.2)	99.8 (99.1–100.0)	<0.001
Uptake of on-site RST, % (95% CI)	92.7 (91.1–94.1)	97.2 (95.5–98.2)	<0.001
Willingness to go clinic for confirmation & treatment, % (95% CI)	56.9 (44.1–68.8)	91.1 (79.3–96.5)	<0.001
Successful referral to STD clinic, % (95% CI)	26.7 (18.0–37.6)	85.7 (74.3–92.6)	<0.001

Out of 1,805 FSWs accessed by outreach teams, 95.2% (95% CI 94.1–96.1%) were willing to get RST on site and 94.2% (95% CI 93.1–95.2%) finally got the testing. Among the FSWs positive for RST, less than two-thirds (71.8%, 95% CI 62.5–79.6%) were willing to go to the STD clinics for confirmation of syphilis diagnosis and treatment of the infection, and 51.9% (43.4–60.3%) went to the STD clinics for further clinical interventions. In comparison with the CDC teams, the RHH teams had more FSWs to be willing to get the RST (99.8% *vs.* 92.8%, p<0.001), take the on-site RST (97.2% *vs.* 92.7%, p<0.001), be willing to go to the STD clinics for confirmation and treatment (91.1% *vs.* 56.9%, p<0.001), and be successfully referred to the clinics (85.7% *vs.* 26.7%, p<0.001), Table 1. Multivariate analyses indicated that FSWs accessed by CDC teams had a significantly lower uptake rate of on-site RST than those accessed by RHH teams (AOR, 0.12; 95% CI, 0.02–0.73; p = 0.02).

## Discussion

Syphilis control endeavors among FSWs and patients at STD clinics in China are crucial because they have a higher incidence of syphilis and are one of the most-at-risk populations for acquisition and transmission of syphilis and HIV. In addition to the risk behavioral interventions, screening for syphilis and treatment of the infections have been equally considered as key strategies in the National Syphilis Prevention and Control Program [4]. To our knowledge, this is the first study in China to evaluate the efficiency of different health providers on delivery of outreach RST services. A highlight of this study is its nature related to the implementation research of medical interventions for syphilis control in FSW settings.

FSWs have become one of the important populations to drive the STI epidemic and probably the bridge population for the heterosexual transmission of HIV and other STIs in China. FSWs in China can be categorized into venue-based and street-based subgroups and the venue-based subgroup can be further divided into those FSWs working in high-class and low-class sex venues. Outreach programs are rather a new development in China to expand HIV/STI services to high-risk settings in order to improve accessibility and acceptability to interventions. Delivering intervention services to FSW population presents various challenges including structural barriers hindering access to care as well as patient's distrust of public clinics [5], [6]. The difference in syphilis seropositivity between FSWs accessed by RHH and CDC outreach teams may be due to that the RHH teams usually covered the FSWs at a higher risk for syphilis infection than the CDC teams. Our findings indicate higher efficiency of the outreach services delivered by RHH teams to address the syphilis testing and referral issues than CDC teams. The relatively high rate of successful referral of FSWs positive for syphilis to the designated STD clinic is mainly because the counseling on reproductive health care and family planning was usually integrated into the routine outreach services delivered by the RHH teams to this population. A study in Southwestern China indicated that although access of FSWs to outreach services was high, awareness of where to access family planning services was relatively low as suggested by a big proportion of FSWs reported having an abortion in the past 6 months [7]. Reproductive health issues are usually serious concerns and big needs for FSWs [8]. In addition to HIV/STD health education and condom promotion, provision of the reproductive health, family planning, and cosmetic counseling and/or services to FSWs is sometimes important to create or strengthen the friendship and/or trust between this population and the outreach members [9], ensuring those women to more likely comply with the advices from the outreach members for risk behavioral change and health care-seeking and achieving a significantly higher uptake of syphilis testing and referral than the current study. However, the health facilities such as reproductive health clinics and community health centers have not been efficiently included or mobilized by the current STD/HIV intervention programs and are usually less invested by public health funds in China. The findings from this study will have important implications for design and implementation of the prevention and intervention programs targeting FSWs.

There are some limitations to be addressed. First, as this study was carried out only in one city located in Southern China, the health system, the socio-economic development, and the health-seeking behaviors of high-risk groups in the study area may be different from other areas in China. Although the FSWs participated in the study came from different types of sex work venues, this population, particularly those FSWs on the street, was not adequately represented in the study sample. Any generalization of the study results should be made with caution. Second, the convenience samples, rather than the random samples, were recruited and some eligible subjects refused to participate in the study, likely resulting in the selection biases. Third, although some FSWs with positive RST were not successfully referred to the designated STD clinics, they might go to other clinics for further confirmation and intervention, likely resulting in underestimations of the successful referral rate.

Based on findings from the study, it can be concluded that RST at sex work venues is well accepted by FSWs when it is integrated into ongoing outreach services. However, successful referral of those FSWs who tested positive for RST at sex work venues to the STD clinics is substantially low. The RHH outreach teams were more efficient than the CDC teams to improve the uptake of syphilis testing at sex work venues and the referral of infected cases to the STD clinics for further medical interventions. In addition, these findings can be helpful for developing the outreach services to FSWs in which rapid HIV testing is planned to be integrated. Regarding efficiency of outreach interventions to FSWs, it may be important, on one hand, to consider the participation of reproductive health and primary health care providers in the services to hard-to-reach population in order to meet the needs of this population. On the other hand, the capacities of the current CDC system in China are needed to strengthen in order to provide more friendly and efficient public health and medical services to this high-risk population. The current health-care reform in China focusing on public health services, medical treatment, and medical insurance will allow re-thinking of organizational and financial models of health providers to actively contribute to the syphilis control program in the country.
